# Chilblains Associated with Chronic Chikungunya

**DOI:** 10.4269/ajtmh.21-0884

**Published:** 2021-10-25

**Authors:** Marcus Villander Barros de Oliveira Sá, Daniel Sá Araújo Lins Carvalho, Luydson Richardson Silva Vasconcelos

**Affiliations:** ^1^ Real Clínica Médica, Real Hospital Português de Beneficência em Pernambuco, Recife-PE, Brazil;; ^2^ Instituto Aggeu Magalhães, Fundação Oswaldo Cruz, Recife-PE, Brazil

**Figure 1.**
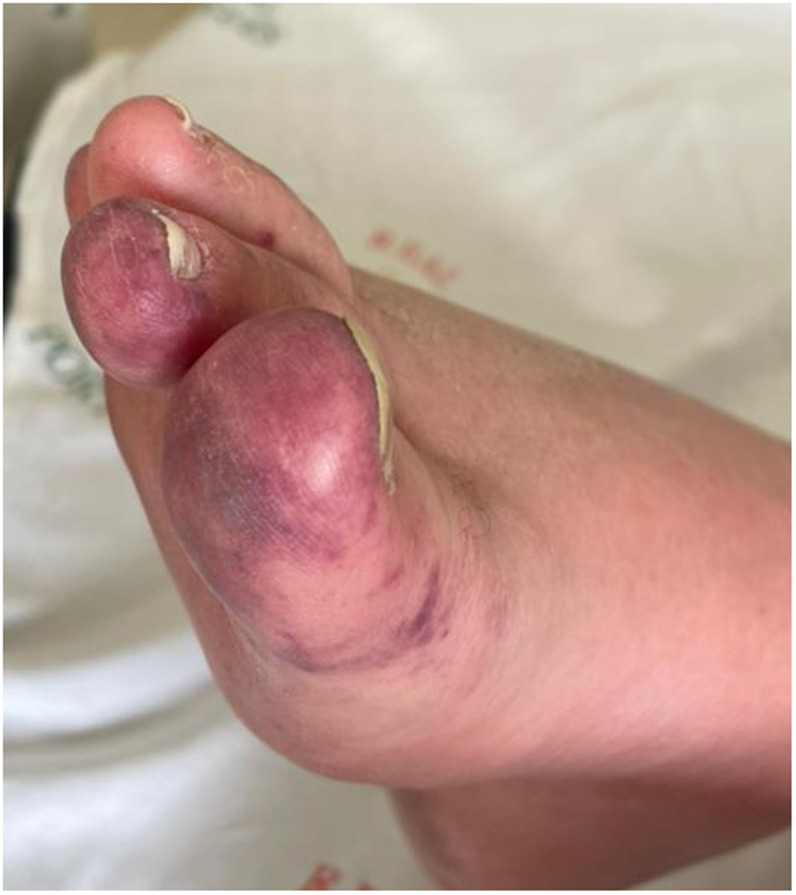
Painful violaceous erythematous skin lesions on toes. This figure appears in color at www.ajtmh.org.

**Figure 2.**
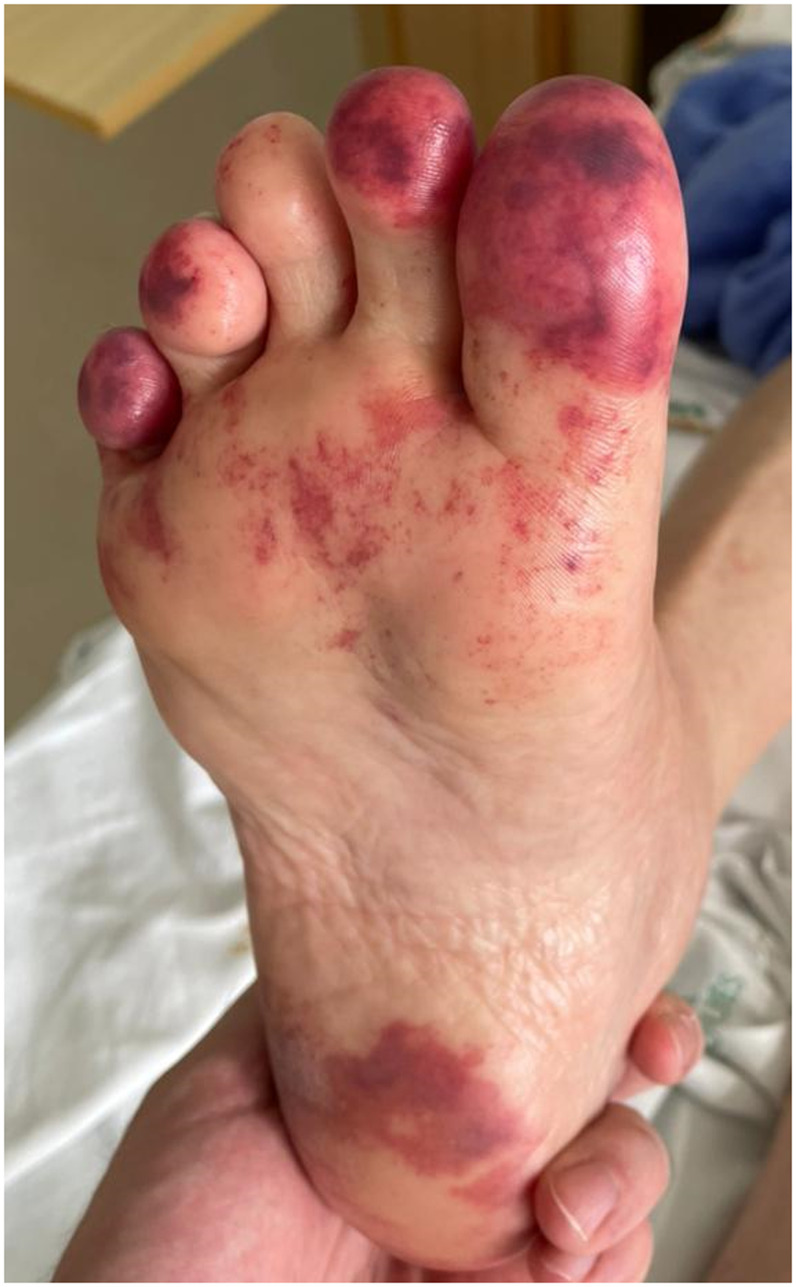
Painful violaceous erythematous skin lesions on feet. This figure appears in color at www.ajtmh.org.

A 63-year-old woman was admitted to the intensive care unit (ICU) because of pulmonary thromboembolism. She had a history of multiple autoimmune diseases as Crohn’s disease, myasthenia gravis, and Hashimoto’s encephalopathy, all of them in remission. She had been on treatment of chronic chikungunya for the past 3 months and was weaning off from steroids with prednisone 10 mg/day and using hydroxychloroquine 400 mg/day. During her ICU stay, she developed a diffuse rash and painful violaceous erythematous skin lesions on the feet (Figures 1 and 2), predominantly in the toes. It was associated with new arthritis in the hands, elbow, and ankles. Antinuclear antibodies, complements, cryoglobulins, antiphospholipid antibody, rheumatoid factor, anti-CCP, parvovirus, EBV, CMV, and serology for HIV, HBV, and HCV were negative. Serology for arboviruses showed positive IgG and negative IgM antibodies for dengue and Zika virus, and persistently positive IgM and positive IgG antibodies for chikungunya, suggesting the hypotheses of chronic chikungunya flare. Doppler ultrasonography of the lower limb arteries and abdominal ultrasonography ruled out peripheral arterial occlusive disease and abdominal aneurysm. Punch biopsy of the lesion revealed red cells in the superficial dermis associated with foci of epidermal necrosis. No signs of vasculitis or thrombosis were found in this sample, suggesting changes secondary to nonspecific vascular damage. She was diagnosed with chilblain lesions related to chikungunya. We hypothesize that chilblain lesion may have been caused by chikungunya. Chikungunya fever is an endemic disease in northeastern Brazil, and its typical clinical presentation is characterized by fever, polyarthralgia (with or without arthritis), and diffuse rash.^1^ Chilblains are characterized by skin lesions (macules, papules, plaques, or nodules) that begin 12 to 24 hours markedly after cold exposure and resolve within a few weeks. Itching, pain, or burning may accompany the lesions. Treatment mainly involves local heating.^2^ The most frequently reported and most studied disease in which chilblains are found is systemic lupus erythematosus (SLE).^3^ There are no pathognomonic histopathological or serological findings to confirm its diagnosis.^4^ The pathogenesis is unclear. Cold-induced vasoconstriction resulting in hypoxia that stimulates an inflammatory response is a potential mechanism.^5^ Given the patient’s extensive list of autoimmune comorbidities, it is hypothesized that chilblains occurred secondary to an immunological phenomenon with vascular repercussion after cold exposure in the ICU as a trigger. She was treated with local heating, prednisone 40 mg/day, and methotrexate 15 mg/week with resolution of the lesions within 30 days.
